# Report on Practical Dentistry, at the Eighth Annual Meeting of the Am. Soc. of Dental Surgeons

**Published:** 1848-10

**Authors:** C. O. Cone

**Affiliations:** Baltimore.


THE AMERICAN

### Journal of Dental Science.

Vol. IX.] OCTOBER, 1848. [No. 1.

### ARTICLE I.

*Report on Practical Dentistry, under the adoption of Resolutions at the Eighth Annual Meeting of the American Society of Dental Surgeons, held at Saratoga Springs, August* 3d, 1847. By C. O. Cone, D. D. S., of Baltimore.

I would beg to introduce the report by a brief examination of the history and doings of the association, that it may present itself confined not only in, and acting in conformity with the spirit and intent of the resolution that gave origin to the same, but discharge the duty of the committee with a strict adherence to the designs of the Society.

This, the oldest surviving dental association in the world, was the third in its rising, of those bright orbs, the light of whose rays are to guide the members, and then the profession, to elevation, learning, and a high established character, in the catalogue of sciences.

Agreeably to a summons from that hand which planted the seeds of its successors, fifteen or twenty members of the profession met in New York, on the eighteenth day of August, 1840, and there formed themselves into the American Society of Dental Surgeons, adopting a constitution and by-laws for their government.

The object of the association is plainly stated in the preamble of the constitution—"to advance the science, (dental surgery,) "by free communication and interchange of sentiments, either written or verbal, and in fine to give character and respectability to the profession, and establish a line of distinction between the truly "educated and uneducated." And in corroboration of this, the adopted constitution prescribes the qualities for candidates, and so far changes the period of preparatory dental education, as to demand of its candidates a studentship of two full years, with some practical dentist known to the Society as such, or a diploma from a regularly chartered Dental College.

This feature of the constitution produced a decided and happy influence on the profession, by bringing into the profession, by increase, members, if not better instructed, with a more mature familiarity with its obligations and duties, thus accomplishing for the profession a double good, by first establishing more generally with the community, an experimental knowledge of the permanent benefits conferred by a judicious practice of the profession; thus elevating it, by rendering the profession an indispensable auxiliary to the health and comfort of society; and the character which will ever be attached by the philosophical mind to any class of men, who demand time and previous education, to enter upon or practice any calling or profession, guided by the light of science.

The by-laws also, granted powers to confer special tokens of approbation on marked improvements in the profession, and established an order for the annual appointment of five of its members, to prepare dissertations on subjects connected with the profession.

We find that in addition to organizing the association at this, the first meeting of the Society, it appointed twelve of its members to prepare tracts on subjects connected with the practice of dental surgery, written for popular and undoctrinated instruction. During the following year, this feature of the Society was carried into operation; and much good was gained by the profession, by its awakening in the community a spirit of inquiry into the profession, its standing and capabilities. Notwithstanding the assertion that this feature of the association was abused, by rendering it subservient to individual, rather than professional prosperity, it is to be regretted that this mode of disseminating correct information among the "sovereign people" should have declined, in the estimation of the Society; for whatever might result to individual benefit in this particular, must also redound to the good of the great whole.

We find, soon after the adjournment of the Society, the avowed objects of the association reiterated by the corresponding secretary, and he adds that the object and operation of the Society will have a "tendency to awaken a new spirit of inquiry and physiological research, both into the theoretical and practical department of the profession, that may result in the increase of its usefulness."* The recording secretary, during the same year, presents to the profession the same objects as those enumerated, and other high, honorable and scientific ends, as being the only aims of the Society's establishment.†

No one who is at all acquainted with the state of the dental profession in the United States, will dispute that its condition has greatly improved since the establishment of this association. Although there were many among the dentists of this country ten years ago, who were men of science, and some who were distinguished for their learning and skill—men whom any profession would have been proud to claim—yet, as a body, the dentists were scarcely entitled to the appellation of a learned profession. There was not any period or prescribed course of study considered necessary, anterior to the establishment of the Baltimore College of Dental Surgery, the encouragement of which, we are told, was a "favorite object" of the association;‡ and nothing was more common than for the young man to leave the plough or work-shop, as he was prompted either by indolence, bodily infirmity or caprice, and, after spending a few months, or more frequently weeks, with some neighboring dentist, pass, without examination, diploma or license, to all the privileges of a regular member of the profession.

The present state of things is far otherwise. So entire is the

* American Journal and Library of Dental Science, vol. i, p. 178.

† Ibid., vol. i, p. 194. ‡ Ibid., vol. i, p. 192.

change of opinion on this subject, not only in the profession, but in the general sentiment of the community, that no young man would be received, at scarcely any point of our country, as a dentist, who cannot produce his testimonials from some established dental institution. Again, it is not the least of the benefits resulting from the change which has been effected, that the profession is no longer answerable in its reputation, for ignorance and misconduct of uneducated pretenders to dental skill. Irregular practitioners we have, and must still continue to have amongst us. But they can no longer mingle in the society of educated and respectable dentists, and identify themselves with them. The line of distinction is so strongly marked between them, that they cannot by any possibility be confounded or mistaken for each other. On the contrary, they are to each other, so far as they are brought into contact, objects of mutual dislike and hostility. The consequence is, that although an irregular practitioner may sometimes so far succeed as to acquire a certain degree of support, yet that support is generally very feeble and precarious; and he can never rise to anything like respectability, and standing in the profession and in the community, be his pretensions what they may, without a regular and acknowledged education, he is still a quack, and can never transfer himself from the class and standing of a quack, to those of a man of science.

The early design of the association was such as to secure the profession in a great measure from the effects of occasional misconduct among its acknowledged members. Dental Colleges may educate and perfect a student in the most accomplished manner, and pronounced, by examination, as exhibiting qualities fitting him for practice; but they cannot pronounce that he will not use those very qualities for purposes that would disgrace the profession; and it would be wrong and ungenerous to expect a faculty of any institution to be held responsible for acts of its graduates, committed independent of the institution. But the Society has full power over its members, to "expel for immoral conduct, malpractice in business, or other sufficient cause."* It cannot, as before intimated, be pretended, nor can it even be expected, that even among those who have complied with the requirements necessary to enter the profession regularly, there will be none whose conduct will be unworthy of the profession. But, although this should be the case, the disgrace will fix its stain upon the guilty individual, and not upon the profession as a body. The very fact that his deeds are a breach of rule, proves that the profession, whose rule he violates, disapprove of the act themselves; while his own consciousness of disgrace, will usually cause him to shrink from those who would feel dishonored by professional recognition. Your reporter cannot forbear, before closing this sentence, to urge the members to constant vigilance in sustaining and exercising this feature of the association.

Another of the objects of the Society, in part gained, and which is being accomplished; is, that it has greatly promoted cordiality and harmony among its members; qualities which the profession have not been proverbial for.

At the second annual meeting of the Society, convened at Philadelphia, the 11th of August, 1841, we find, in conformity with appointment, papers read of the first order; those that were more than equal with anything that had ever before appeared in dental literature. The Society at this meeting assumed the charge and supervision of the American Journal and Library of Dental Science, and appointed two editors to superintend and conduct its publication. The Society did not design engaging in book-making, but intended the Journal to contain the transactions of the Society, and the communications made to it, in form of essays, from time to time, and the current literature of the profession. The Library was intended to rescue from oblivion old and standard works, from our own, as well as other languages; and we may now say that the American Journal and Library of Dental Science, comparatively speaking, contains *the* literature of the profession.

We find the Society, at this meeting, thus early taking a

*American Journal and Library of Dental Science, vol. i, p. 160, and vol. vii, p. 96.

stand of authoritative, but just independence and supervision over the practice of the profession; by pronouncing certain indulgences by the profession malpractice.

The third annual meeting of the Society was held at Boston, commencing the 19th day of July, 1842. We find again, in the published proceedings of the Society the literature of the profession increased by the Opening Address, by Dr. E. Parmly. An Essay on the Diseases of the Maxillary Sinus, by Prof. C. A. Harris, a paper more comprehensive than any that had ever appeared on this subject, filling a vacuum in medical and dental pathology, that had long existed; a paper by Dr. J. S. Gunnell, directing the practical means to be used for reducing accidental or congenital protrusion of the inferior maxillary; another paper by Dr. Solyman Brown, on the "Surest Means for Degrading the Dental Profession;" which was clothed in the severest ironical language, showing the means employed by the irregular portion of the profession to establish a position in the estimation of the community. The number of the Society was increased by a large number of admissions at this meeting, and the application of a greater number, laid over for future consideration.

The Society seems at this meeting to have taken alarm, either from the consciousness of having already admitted to membership, either by haste or accident, such as were not in accordance with the design of the association, or from the large number of applicants at this meeting; and established local committees, to examine into the justice, claims and professional standing of candidates for admission. The action of these local committees seems to have been small, and eventually abolished by the Society, whether from a want of activity and enterprise of the members that composed them, or the more probable cause of inefficiency for the purpose for which they were constituted, by the difficulty the committees experienced of frequently being in consultation, from the remoteness of their residences from each other; cannot be fully determined.

We find at this meeting a growing liberality shown, by some of its members making public exhibition of dental instruments of recent invention or application. Your report would take this opportunity, to urgently impress the members of this association with an active desire to cultivate this spirit of liberality, by each member bringing some offering, however trifling; let it be brought at each annual meeting, and laid on the altar of inspection and examination, as a tribute of sincerity, of professional liberality and good feeling.

At this meeting, the attention of the Society was occupied with papers that related to alleged violation of professional etiquette, on the part of members of the association. This feature of the association—the establishment of a tribunal for the adjustment of differences among its members, cannot but have had a powerful effect in preventing them. Although, since this meeting, rarely, if ever, has it been appealed to for this purpose; still the general diffusion of sound learning has been a strong auxiliary in sustaining this operation of the Society, and lightening its duties, by promoting good fellowship among its members and the profession generally, by leading them to found their expectations of success upon scientific acquirements and true worth, rather than upon those acts which are the recourse of the ignorant, and the never-failing source of jealousy and discord.

The leading object of the organization was the promotion and diffusion of dental science among the profession; and this object has been steadily kept in view in all its proceedings, and it has been in no small degree successfully accomplished; and the report hazards nothing in saying that the profession in general are much better instructed since the establishment of the association, and their patients more skilfully attended than they would have been at this time, if the Society had not been instituted.

The report does not claim for the Society the direct agency, or exclusive praise for these important improvemets in the profession. The general advancement of learning and education has done much, but the dental colleges have done more, by greatly increasing the means of acquiring professional knowledge. But the Society has been a powerful auxiliary to this end, by becoming the patron to the dental colleges; since it extended the demand for instruction by the necessities it imposed on members and candidates. This is one of the peculiar provinces of the association, and its powers in this respect are ample, and should be most rigorously, faithfully and perseveringly executed.

The fourth annual meeting of the association was held at Baltimore on the 18th of July, 1843. The opening address was delivered by Prof. C. A. Harris, in which he gives an exposition of the object and doings of the association, its ends and design, in graceful and dignified language. There was a paper presented by Prof. Thos. E. Bond, Jr., at the request of the Society, on the morbid sympathy between the mouth and other parts of the body. The request just referred to, showed plainly the anxiety of the association to extend the knowledge of general pathology in the profession. A paper was also read by Dr. Amos Westcott, on dental caries, embracing a series of physiological experiments, that confirmed, established and explained important principles of dental pathology. A paper of some merit was also read by Dr. J. S. Gunnell, on the painful cutting of the dens sapientiæ.

The fifth annual meeting of the Society was held at New York, the 17th of July, 1844. Two papers appear among its published proceedings, as having been read. One, the claims of medical science on the dental practitioner and student, by Dr. Amos Westcott, in which the author labors to, and proves the absolute necessity of the dental practitioner being familiar with the numerous branches which constitute the whole of medicine. This paper was little more than an explicit and faithful expose of the spirit of the association—its demands and expectations of the profession. The other paper was read by Dr. W. H. Dwinelle, which related to physiological consideration of that substance known as salivary calculus.

The Society at this time made some slight alterations in the constitution, and barred a little more strongly the avenues of entrance to membership. It also had under consideration the subject of the formation of state societies, to be rendered auxiliary to the American Society of Dental Surgeons; but before a carefully prepared plan for such associations was reported by the committee appointed for that purpose; which would have been so formed as to harmonize with the simplicity of operation, elevation of purpose, and firmness of action of this association, other state societies were formed, absolving the Society from any future action on that point.

The sixth annual meeting was held at New York, on the 5th of August, 1845. A number of papers were read before the Society. One, by Dr. E. J. Dunning, contained some excellent hints to the dental practitioner, although the main subject he attempted to prove is still a question open for discussion. Other papers were read by Drs. E. Parmly, E. Townsend, John Allen and James Taylor; all of which, with the exception of some remarks by Dr. Parmly, which relate to practical points and features in the workings of the association; partook more of the character of popular addresses, than thorough discussions, of purely scientific subjects. Much of the time of the Society, at this meeting, was taken up in endeavoring to ascertain how far its members practiced in accordance with the expressed sentiments of the association; and it adopted some caustic resolutions, and imperative demands of its members, and penalties in case of refusal.

The action of the Society in this particular was censured by many well-wishers of the profession. But it can easily be seen, when dispassionately and impartially examined, as being not only an act of justice, but one of right and power. The Society, at the first opportunity that was offered to express sentiments on special points of practice—at its second annual meeting—placed their seal of disapprobation on the use, by the profession, of all substances for filling teeth, composed in any part of mercury,* in such terms as could not be mistaken; and it cannot but be admitted, that the founders and early builders of the association looked upon a measure that should suppress this mode of practice, as a favorite and early scheme.

In a review of the doings of this meeting of the association,

* American Journal and Library of Dental Science, vol. ii, p. 136.

by an official member, we find him using language highly approbatory of the course of the Society,* in dismissing itself from further action in securing legislative protection for the profession, or legal favoritism for the association. Such a course, if adopted by the Society, if it would not have been in opposition to the workings of the bodily members of the association, was in direct contradiction to the spirit and soul of the institution. The design of the Socity never has been to confer privileges upon its members, and in no way distinguish them from other practitioners, except as it gave testimony to the fact that they had voluntarily submitted to the requisitions of the association, and met its approbation.

The principle to which the American Society of Dental Surgeons must look for efficacy, in gaining the end and objects for which its course has been steadily designed, must be gained by familiarizing the community with its workings; and the confidence placed in it by the professional and unprofessional, that its officers will not, on the one hand, give countenance to any dental practitioner who has not acquired such a practical and theoretical education, and moral qualities as to entitle him to support; nor, on the other hand, withhold it from any who are thus qualified, and will sustain its truth of avowal, and expel, if it be necessary to retain its purity, even to its last member. This will be, and is, the true secret, if it may be called such, of the Society's power.

At this meeting, the Society again revived that important feature, of distributing dental information among the community, by publishing short aphorisms on the most important subjects of dental practice. But the future action of the Society on this subject, as provided by the resolutions which appointed thirteen of its members to prepare these aphorisms, in the excitement of the succeeding meetings, appears to have been lost sight of, or at least failed to be acted on.

This meeting resolved to, and did disseminate some of its doctrines and opinions, through that powerful agent, the public

*American Journal and Library of Dental Science, vol. vi, pp. 68 and 69.

press; a course which it is hoped the Society will not forget soon to follow again.

We again find the members of the Society examining the construction and philosophical applicability of dental instruments. The Society seems to have fully felt the effort of the exhibitor to keep in mind the duty and design of the institution in this particular, by tendering to the liberal designer and executor, Dr. W. H. Elliot, the thanks of its members.

This meeting closed after setting apart a portion of time of each annual convocation, for the oral discussion of some proposed subject of dental practice. But to the regret of its members, this important design was not adhered to, although it has not been lost sight of entirely, and swallowed up in the more exciting and distracting business that engaged the attention of the succeeding meetings of the Society. That the oral discussion of practical subjects, which shall have been previously determined on, will be attended with most positive benefit to members of the Society, cannot for a moment admit of question. It will present to the members of the profession that information which would never find its way into the text books or literature of the profession; as before intimated, this design is not dead but paralytic, and this meeting of the association is looked to for electrifying it into renewed and pristine vigor.

During this, as well as the previous and following years, many new names were added as contributors to the literature of the profession, which was published in the American Journal and Library of Dental Science, the organ of the Society. Its honorary members and foreign correspondent furnished a number of lengthy papers, all of which were most valuable acquisitions to the profession, but of course possessing in their characters exceedingly various degrees of excellence.

The seventh annual meeting of the association met in New York, on the fourth day of August, 1846. The first business that engaged the association was to examine into some empirical proceedings on the part of some of its members, and ascertain the compliance of the same with its demands. The course of the Society was one of hesitation in dealing with such of its members as had signified their unwillingness, to tender their submission to a demand of the association, which was given expression to on the *first opportunity* that was presented after the organization of the institution. Some of the members who now opposed the tone the Society was compelled to sustain, or depart from its original design, took part and voice in its early action in relation to the subject of dispute; while others, entered the Society with the *prima facie* evidence of its demands standing in *bold relief* upon the published doings of the association.

The vacillation of the Society upon this subject, could only be excused by the recollection of a laudable desire on the part of the association to act with caution, first being in full possession and examination of facts. Had the Society pursued a less absolute course, or dealt less severely with its revolting members than it did, at its next meeting, however unpleasant to individual feeling, by thinning its ranks by their expulsion, it would have resulted in lowering the dignity of the association, weakening its powers, and neutralizing its end and object. In its government and workings, the Society must ever be, to attain its end, as uncompromising as justice.

At this meeting two papers were read, one by Dr. W. H. Elliot, and the other by Dr. W. H. Dwinelle; both of which contained some valuable points of practical consideration.

The constitution at this meeting was materially changed. Another step of advancement, which the Society had steadily pursued, was taken, to more effectually secure itself against the entrance of members not qualified for its high professional association, by demanding that "All applicants for membership to this Society shall be at least twenty-one years of age, shall have a good English education, shall produce evidence of unexceptionable moral character, and shall have studied and practiced for the full term of two years, in a regularly chartered dental college in any of the United States, or with some respectable dental practitioner, known as such to this Society, and have practiced, in addition, as a dental surgeon, for the full end and term of three years subsequent to the above-mentioned term of instruction; or, in lieu of the aforesaid five years of studentship and practice, he shall have practiced as a dental surgeon for the full term of six years." * * * * *

"Application for membership, as acting members of the Society, shall be in writing, and recommended by at least three members, who are personally acquainted with the professional qualifications and moral character of the applicant, and are willing to vouch for the same; they shall be made to the secretary of the executive committee, at least six months previous to a regular annual session. All propositions for election of *honorary* members shall be signed by at least three members of this Society, who are willing to vouch for the professional qualifications of the persons so proposed, and shall be addressed to the secretary of the executive committee. The said committee shall, if possible, investigate the subject immediately, and report to the Society as soon as practicable; and the candidate may be balloted for at any regular annual session, as soon as the committee report."

"No application for active membership in this Society shall be considered, until the full term of six months after the application shall have been received by the executive committee, in the manner described" in the foregoing quoted sentences, "and at no other time than a regular annual session. Propositions for membership, as honorary members, may be considered as soon as the report of the executive committee upon such proposition has been received by the Society, which cannot be acted upon except at a regular annual session."*

Thus it is seen above, that if the Society, by accident or imposition, held in communion such as were not worthy, it was fully determined that it should not longer offer means of further admission of such, but like the pure liquor, which had thrown off the scum and filth with which it was early mixed, should be replaced by that which is pure in quality, to ripen on its lees.

A resolution was adopted at this meeting, to prepare a code of ethics; and it is to be regretted that the committee to whom this duty was assigned, has not yet been able to complete their labors and report. That a properly arranged code of ethics,

*American Journal and Library of Dental Science, vol. vii, p. 96.

embracing the duties of the dentist to his patients, of patients to the dentist; the duties of dentists to each other, and particularly to members of this Society; the obligations of the profession to the public, and the public to them—would guide the action of the members in consummating the end of the Society, the elevation of the profession. In this connection, the report would draw the attention of the Society to the consideration of adopting some clause or feature to such a code of ethics, that shall determine the members when and with whom to exchange professional courtesies.

At this meeting, the first step was taken in that effort which was to give origin to your committee, by adopting the following resolutions, viz.

"Whereas, it being an admitted truth, that experience, aided by observation and comparison, has been the main object in establishing facts which have rendered the utility of dental surgery such as to deserve the gratitude of mankind, and elevate it to the dignity of a respectable, learned and responsible profession; and it being one of the avowed objects of this Society to to render such facilities as it shall be enabled to, to multiply such facts, "the advancement of the science," (dental surgery,) 'by free communication and interchange of sentiment,' and believing that the object of the Society upon this subject will be more fully carried out by some systematic course,"

"*Resolved*, That this Society appoint a committee to prepare and report to the Society a tabular sheet, to be used by its members; to make an annual report to the Society of all their operations, and the characteristics of the teeth and mouth upon which such operations are performed, together with a detailed description of the treatment and preparation of the mouth for, and the application of, all artificial pieces; together with the previous failures in either surgical or mechanical dentistry that may come under their treatment, together with what, in their judgment, they consider the cause of such failure."*

Up to the time of this meeting, and even later, the Society has found its active opposers, and those who felt indifferent to

*American Journal and Library of Dental Science, vol. vii, p. 100.

its success; and these, even among some of the better portion of the profession. This state of the profession is not to be looked upon with wonder, when we recollect how recently the profession existed only individually. Many felt unwilling to associate with a Society, lest they should degrade their own high qualities; others saw, with similar eyes, some one of the Society's members, who did not rightfully belong in such an association, and failed to pursue a wise course, by uniting with the members of the institution to reduce such to their true level. Others, conscious of the purity of their own designs, forget the truth of the maxim, that there is "strength in union," and trusted to their efforts to elevate and determine the profession by individual effort.

If to some, the Society has shown at times apparent irregularities in its workings, or a disposition to make it subservient to individual interest and localize its acts—or held in union crude material—or failed to interest some influential and highly qualified members of the profession in its welfare; then let it be remarked that the simplicity of the workings of the association has ever been preserved in the main, and that its course to elevate the profession, which was its end, has been steadily pursued, and in no small degree attained. The Society has been made up, not only at its organization, but by increase, of the worthy, respectable, and the majority of the most eminent practitioners in this country, but who were little accustomed to act in concert, and little aware of the full extent of their duties, or extent of power which a combined action would give to the profession, without doing violence to the rights of any. Considerable time was thus consumed, while the members became accustomed to their new privileges and duties. But nothing is risked in the assertion, that the association has accomplished as much for the profession as any similar society, for *any science*, in the same length of time.

The eighth annual meeting of the Society was held on the 3d of August, 1847, at Saratoga Springs. The early part of the session was occupied in dismissing from membership a number who refused to comply with the requirements of the Society.

There was only one paper read at this session of the association. It was from Dr. Wm. H. Elliot, on the subject of "absorption of dental bone," (referring to the fangs of the temporary teeth.) The physiological deductions made from the cases which he relates in the paper, might be used with the same advantage to sustain the opposite side of the physiological subject of discussion, as the position taken in this essay. The facts, or cases brought to prove the position of the writer, are too few, and their evidence to the point he desires them to sustain, too contradictory to admit of judgment being pronounced by the profession. It may be reasonable to suppose, however, that the writer had other evidence like that presented to establish his position; but such proof must come in to sustain his position, more as collateral evidence, and after ocular demonstration, aided by the knife, has satisfied the experimenter of the absence of Bourdet's "absorbing apparel," which that dental physiologist so carefully describes, by the assistance of anatomical dissection, and affirms, works the destruction of the fangs of the temporary teeth. It is hoped the subject now under consideration will not be neglected by the author of this paper. The members of the Society will anxiously look for further proofs, either to establish or deny the position assumed by the writer; and there are few in the profession better qualified, by an ingenious and patient mind, for this duty, and to lead the profession to other and more extended physiological researches, by the successful termination of his labors.

The committee appointed to prepare a tabular sheet for the use of members of the Society, to record the aggregate facts of their annual practice, made their report. The importance of this measure cannot be overlooked, particularly when viewed in connection with your committee, for whose assistance and wants it was intended to meet and anticipate.

The measure or its importance is neither novel or impracticable. Over two hundred years ago, Lord Bacon used the following language in relation to recorded facts: "The understanding is as incapable of acting on such material with the aid of memory alone, as any person would be of retaining and achieving by memory, the computation of an almanac. We cannot approve of any mode of discovery without writing, and when that comes into general use we have further hopes." And a little further on, he continues: "We shall then be able to put the forces of the understanding in due order and array, by means of proper and well arranged, and, as it were, living tables of discovery of those matters which are the subject of investigation, and the mind can then apply itself to the ready prepared and digested aid which *such tables* afford."

The science of medicine, in *all* its branches, is made up of simple facts, ascertained by observation and experience; and it is in these that the profession of dental surgery is deficient, and which it demands to give it an established and permanent standing as a profession and science.

It is from a simple collection of facts, that the whole diagnosis of the medical profession is based. But a short time ago, and it was not considered possible for the medical practitioner to distinguish *pleurisy* from *pneumonia.* Formerly many cerebral diseases were all embraced under the common division of inflammation of the brain, which are now known to be distinct affections; and so of pulmonary and cardic diseases and fevers, which have received divisions which experience and truth have established, and led not only to a better and more correct knowledge of the pathology of disease, but to their more successful treatment, by a simple collection of facts. The only contribution to this end, ever made through the medium of the Society,* if it has not produced as great and decided beneficial marks on the profession, has, nevertheless, tended to establish the doctrine and pathology of dental caries, and improved the knowledge of the profession. Yet upon this very subject much is still to be explained and made known.

It is a principle of the human mind, to seek and determine a cause for every circumstance or fact that may frequently occur, or be presented to human observation; and it is equally true, that the cause so determined is more frequently wrong than right. Unless we make a written history of the case, we gather

* American Journal and Library of Dental Science, vol. iv, p. 31.

and recollect only such facts of the case in question, as may coincide with impressions or prejudices; and, moreover, we are unable, from memory alone, to make that careful and close analysis of the different features which a case or cases present, to establish scientific and correct conclusions. Hence it is, that so many *false facts* are established, and considered invulnerable; to the exclusion of more simple and unostentatious truths.

The applicability of this method of procuring information, proposed by the Society, seems to be peculiarly adapted to the practice of the dental profession. No branch of the healing art is more definite in its practice, or more plainly presented to the senses than dental surgery; for it has less to do as an entire profession or a whole, (although by no means exclusively,) with that which is not subject to changes, governed by vivifying principles, not susceptible of explanation. Few of its practices or results are not capable of comparison with some familiar color or hue, or explained by some known fluid or solid, and measured by weight and capacity.

If it be urged that this measure does not promise to bring immediate and direct aid to the practice of the profession, (an admission which will not be granted,) by furnishing new means of exercising the duties of the profession, that would not be a sufficient argument to defeat or embarrass the character of the measure. It is generally known, as well out of the profession as in, that gold is heavier than water; but is there no good growing out of the ascertained fact that its specific weight is 19.25 heavier than water? It is generally known that the pulse of an infant is more rapid than an adult's; but is there no benefit to be derived from the ascertainment of facts, collected similarly to those proposed in this measure; that the average rate of the former is 95, while the latter is 75 per minute? In like manner, would there be no benefit accruing to the profession, if no other facts were to be established, than that dental caries held a certain average frequency in one part of an organ, or of one class, over another? Would there be no benefit resulting from the ascertained fact that the average decay of the teeth of females was in a certain proportion greater than that of males? Would the physiological and pathological fact be of no value to the profession, to know that the teeth and gums of females were more liable to attacks of disease after the age of puberty; and this liability greatly increased during the period of gestation and parturition, than at any other period of life? The proposition is so plain that it needs not argument to establish it. And it is confidently believed that this measure, together with its auxiliaries, which is or may be established, will not hold an inferior position to any that has ever been undertaken to elevate dental surgery to the dignity of a science. It will supply the wants and deficiencies of the profession, by furnishing statistical facts, which has marked the literature by great poverty.

Again, this measure presents other points of urgency in connection with the foregoing. It has a tendency to institute a more careful and analytical mode of thought into the science, and create and cultivate physiological and pathological researches. The profession contains many excellent mechanical practitioners, but too few scientific cultivators of this branch of the healing art. To comply with the demands of this measure, it will require the members of the Society to record their cases of practice, so as to present their facts in the tabular sheet; which must lead to a more careful examination of each case, and the practice adopted; and furnish easy and ready means of comparison, through the medium of the eye, less liable to inflict either self-deception or professional query of accuracy; as one writer quaintly observes, that we might "more readily expect a man to assert a falsehood than commit a forgery." And Bordeu's quoted language, expresses the same in less laconic and caustic terms, when he says, "If every one were obliged to produce the written proofs of his observations, since we too readily adopt the opinions of others, and nothing is easier than self deception," fewer errors would occur. A little further, and he observes, "The observer who is able to furnish well-recorded cases, must not be satisfied with saying, I have seen, I have done, such a thing; for mere stone-blind observers may say as much; he must be ready to convince even a sceptic, who may say to him, pray where did you observe? How did you observe? And, what is of still more consequence, what right had you to observe? What precaution did you take against deception? What proofs had you that you were not deceived?

For the greatest good to accrue to a profession from an adherence to the above principles, it is necessary that a large collection of facts be made, and from a large and extended circle. Two hundred cases, setting forth the same features, in the same or similar circumstances of life, age, &c., might with propriety establish the presumption that two hundred more, under similar circumstances, would present similar results. This expectation gave origin to the measures adopted by the Society, to the end that the circumstances might not only be numerous, but the cases correspondingly so, that comparison in this as well as other particulars might be instituted.

Immediately in connection with the design and object of the measure above referred to, the Society at this, the eighth meeting, adopted the following resolutions, viz.

"*Resolved*, That the American Society of Dental Surgeons shall, at its annual meetings, appoint a committee of five of its active members, who shall be known as a Committee of Practical Dentistry, the duty of which shall be to make an annual report to the Society of the improvements effected in this country in the management of disease coming within the scope of the dental practitioner, and the condition and progress of dental knowledge in America during the year of their service.

"*And be it further resolved*, That it shall be the duty of each member of this association, to communicate all information, or novel results in practice, at their earliest possible convenience, to the above committee, that the above resolutions may be more fully carried out."

These resolutions gave birth to your committee, who enter on their duty of preparing a digest of material which has accumulated during the past year, with little or no available means at their hands, with which to commence their labor. The tabular sheet, which would have furnished sufficient matter for an extended report, was not distributed to the members of the Society, for reasons which it is understood will be made known to the Society by the proper authority. Hence the report your committee proposes to present will be but little more than the commencement of their labors. Yet, with these and other embarrassing circumstances to contend with, your reporter will proceed to the fulfilment of their duty, guided by a sense of the wisdom of the motto of Rosseau, "that facts exist not in the minds of men, but in things and nature."

The report would respectfully draw the attention of the Society to the propriety as well as importance in the design of more perfectly carrying out the object of your committee with greater accuracy in future, to appoint distinct committees, consisting of two or more, upon all the more important subjects connected with dental practice; and your reporter would suggest the following divisions, subject to other additions that may be proposed, namely, a committee to examine and report on the subject of dental and surgical instruments, as employed or relating to the practice of dental surgery; a committee on surgery proper, as practiced by the dental *surgeon;* a committee on dental practice, relating to such operations as are demanded by the living dental organs; a committee to note and suggest improvements relating to any department of mechanical dentistry; a committee to examine and report in relation to the subject of dental physiology and pathology; and a committee to examine and report on the current dental literature, and the state and progress of dental education. The duties of these committees shall not only consist in making reports annually, of the progress of the science in the various branches over which they preside, but to institute and carry on experiments, and report accordingly, from time to time, as the Society may see fit to instruct.

Great caution and judgment should be observed in determining these committees. They should be composed of men peculiarly prepared for their duty, from previous mode of thought and qualifications—men of industry and energy, men of correct judgment, for it is an established truth, that there are as few good observers as good reasoners; in fine, let the question of whom they shall be composed, be answered by, who are the working members? rather than, who shall have the position. To this end, should the Society see fit to take the proposed measure into favorable consideration, and appoint such committees, and give the nominating power into the hands of the president of the association, to be sanctioned by the voice of the members, agreeably to the constitution and by-laws.

The utility of such a measure, the report does not deem it necessary to urge upon the Society further than to say, that it will tend to relieve the Society from the accusation of failing to deliberate and carefully examine, in a scientific and philosophical manner, subjects of importance to the profession. If the Society sincerely desires to add permanent and stable support in elevating the profession, it must be done through these or similar measures.

In considering the subject of dental physiology, the report would desire to engage the attention of the association, with an examination of some physiological considerations connected with that interesting subject,

### *Third Dentition.*

The explanation given of third dentition, by anatomiical and physiological writers, has always been marked by great vagueness and uncertainty; and its actual occurrence, even more than questioned by some. Those that admitted the existence, and attempted an explanation of this developmental phenomenon, attributed it to a freak of nature, unexplained by any law of development.

It is not deemed necessary, in connection with the subject, to establish by reference to facts, the frequent existence of third dentition, by the recital of cases of undoubted, and not to be mistaken development of one or more teeth of third dentition.

Prof. C. A. Harris, in an examination of this subject, since the last annual meeting of this association, has offered an explanation not only of the cause, but also the process by which the cause produces the effect of third dentition; and in full conformity with physiological laws and anatomical development.*

Anterior to the publication of the article of Prof. Harris on this subject, all writers who admitted the possibility of the occurrence of third dentition, referred its cause to a triple development of dental papillæ, which must be referred back to fetal organization.† This doctrine, however, is in direct contradiction to the explanation given by Prof. Harris in the paper under consideration.

He endorses the investigations of Mr. Goodsir, whose examinations in relation to the development of the teeth, commenced at the sixth week after conception, at which period all that presented itself to the eyes of the investigator, at that part of the fetus that was to mark the future locality of the dental germ, was a groove, lined by the mucous membrane. A little later in the period of fetal existence, a dental germ makes its appearance, rising from the mucous membrane, which was said to line the floor of this groove. Still a little later, and the germ is enveloped in a follicle; and, at about the thirteenth week, the germ or papilla has increased in size, and assumed the shape of the tooth which it represents; and about this period the follicle becomes a closed sac,‡ after which it passes through its various stages of ossification, &c., and finally its eruption.

From the above stated investigations of Mr. Goodsir, Prof. Harris argues, that the presence of mucous membrane is necessary for the development of the tooth papilla, and that the position and location of a tissue or part of the organized frame, determines its office and function; as, for illustration, from a fluid of apparently uniform composition, in one place muscle should be formed from it, and in another brain, glandular or ossific matter; and hence the peculiar result attending the mucous lining of the groove described at the sixth week of fetal existence, by Mr. Goodsir. From this, Prof. Harris infers that

*American Journal and Library of Dental Science, vol. viii, p. 116.

† Blake, Bell, Delabarre, Desirabode.

‡ Edinburg Medical and Surgical Journal, January No., 1839.

the presence of healthy mucous membrane in this position is a demand for the appearance of a tooth papilla, whilst its presence, in its turn, makes a demand for its natural appendages, of follicle, &c., which, in conformity with the laws of development, is answered.

When a tooth has been extracted, and the granulated walls of its socket encloses a portion of mucous membrane, its presence at this anatomical location, and in this position, changes its function, and, in accordance with the above deductions, third dentition is the result.

That this explanation of third dentition is positively satisfactory, the report is unable to pronounce; but that it has strong grounds, entitling it to the position of credence, cannot be denied. By this explanation we are able to assign a cause readily, not only for the eruption of supernumerary teeth, but for the cases which are related of fourth, fifth,* and even any number of dentitions. It explains this "freak of nature or accident," as being dependent on laws of organization; by plain and simple principles of developmental anatomy.

Both of the points on which this explanation rests, find support when we examine the contents of some of the abnormal formations known as tumors. That class or group of encysted tumors, the contents of the cysts of which are not serous, occasionally contain highly organized structures, as, for instance, hair, bone and teeth. Whenever teeth have been found as a part of their contents, hair has always been observed. The hairs are frequently found loose in the cavity of the tumor, probably having been thrown off, like epithelial scales, but more frequently, when attached by their bulbs, growing from the lining membrane. In a case related by Prof. J. B. S. Jackson, M. D., the lining membrane from which the hairs grew, consisted apparently of well formed cutis and cuticle.† The origin of these productions in this location, we have nothing further to do with at this time, than to observe that the tumors contain

*American Journal and Library of Dental Science, vol. viii, p. 204.

† Descriptive Catalogue of the Anatomical Museum of the Boston Society for Medical Improvement.

all that would be demanded to sustain Prof. Harris in his position, viz. *mucous*, dermoid and fibrous tissue,* capable of generating teeth, hair and bone.

Sir Benj. Broidie saw a tumor that contained a jaw with full grown teeth; and in almost all cases where teeth have been found, they have been implanted in the fragments of bone or cartilaginous matter, and have resembled the rudiments of maxillary bone and alveolus.† Meckel thinks that those accidental teeth are produced like ordinary teeth;‡ but without inquiring into the history of their formation, we have enough facts to serve the present discussion, to know that in all cases where teeth are found in tumors, there is found mucous tissue, and that they are always enclosed in bone, alveolus, or cartilage, which offers proof to sustain the other position assumed by Prof. Harris, that it is necessary for the mucous membrane to become located in that peculiar position which demands a new action, for a physiological change to take place, which will render it capable of fulfilling the functions and office which its position will determine, agreeably to the law of development.

It might be asked, if the position of this physiological question was established, might we not argue pathologically, and reasonably expect similar results to attend the introduction of mucous tissue into the socket of an extracted tooth, and uniting its mouth or walls; as that which attends accident? And if such a result does not attend such an experiment, would it not be satisfactory proof of the fallacy of this explanation of third dentition?

To these questions your report would reply, that if the presence of mucous tissue enclosed in the socket of an extracted tooth, by its granulated walls, is all that nature demands, to institute a physiological change, which would produce from it a tooth germ; then might we hope, and even expect, to be the agents in supplying the loss of dental organs by the means above referred to, for the reproduction of successors. But suppose such

* Stillé's General Pathology, p. 454.

† Cyclopedia of Practical Medicine, vol. iii, p. 447.

‡ Meckel's Anatomy.

an experiment failed, it would not destroy the truth and establish the falsity of the reasoning and explanation of this phenomenon, only so far as this, that the presence alone of mucous tissue in this peculiar position, and under these peculiar circumstances, is not sufficient to institute a physiological change, which would develop from the surface of the mucous tissue thus circumstanced, a tooth germ; but would tend to prove and establish the fact, that mucous tissue is the parent structure of dental germs; and the presence of mucous membrane in this particular anatomical position, and under the peculiar circumstances described by Prof. Harris, is necessary; but that it also demands a certain idiosyncracy of organization or nutrition, at the time, for the mucous tissue to be capable of producing the tooth papilla; but where the first step in the change towards reproduction has been taken, its successors in the journey of perfection follow, agreeably to the laws of organic, and anatomical developmnnt.

The report would now desire to offer some remarks for the consideration of the association, in reference to that most important duty of the dental practitioner—the preservation of the natural teeth, by the operation generally known as

### *Plugging the Teeth.*

This operation is more frequently attempted—by all—from the merest mechanical dental *tyro* who proffers interference with dental disease, to the accomplished and highly professionally educated dental practitioner; than all other duties demanding the dentist's attention, not even excepting the common operation of extraction; and the failure of the operation from incompleteness of its various stages, is more than equal to a comparison with its accompanying frequency.

Much of the repetition of failure attending this operation, as relates to permanency, even with the better class of practitioners, is often dependent on a faulty judgment, in determining the indications of success attending the proposed operation; the assertion presuming that the operator is familiar with the economy of the human structure and its operations, which shall enable him to exercise his judgment in relation to the propriety of this or any other surgical operation that proposes to restore to health or function, a part of the human fabric; for without such knowledge, the most positive uncertainty attends all parts of such an operator's efforts in this department of the dentist's duties, let his qualities as a mechanical operator be of a high grade; although, by the assistance of the latter, he might succeed in performing an operation which would be determined on, in the absence of the counsel of a scientific education, and prove successful even in spite of nature, and the violation of her laws; which a dentist of scientific capabilities would not hesitate for a moment to discard. Such cases of success are the result of chance and accident, which the man of science never trusts, either the case which is dependent on his skill, or his reputation to; as they answer not back the test of *facts* and *truths.* In like manner, the result is the same, if the operator is deficient in any department of that nice mechanical tact that is always demanded, and on which so much of manual surgery rests in its successful dispensation. Hence it does not become difficult to determine and assert, that the operation under consideration cannot be uniformly well and successfully performed, except by such as possess all the requisites for constituting an accomplished surgeon, and after a long period in the exercise of those capabilities in this peculiar branch or department of the curative art.

It is not considered, by the report, in keeping with the design of this paper, to enter into, or more than briefly repeat, some of the general directions given for the performance of the operation under consideration; further than the examination of some especial feature connected with the operation, demanded in connection with some peculiar state of the organ or case.

After an examination of a tooth, and the result determines the operator, that the extent of the disease will warrant and permit an attempt to restore the organ to health; after properly constructed instruments have been provided and selected for the various stages of the operation; there can be no compromise or reference made in relation to ***time***, ***material***, or ***necessary*** pains to effect the faithful execution of the self-imposed duty, and nothing short of absolute perfection in each step of the operation, before proceeding to the next, will insure uniform, permanent and useful results.

To secure this end, if the cavity or position of the decay is not such as gives a free and smooth surface, as is rarely ever attained without artificial assistance, especially on the approximate surface of teeth much decayed, a file should be used, not only until sufficient strength of edge to the cavity is attained, by healthy dentine being exposed; but an even, smooth surface given to the whole of that portion of the tooth that surrounds and embraces the proposed operation. The instruments employed for this operation, should be so used as to secure the easy escape of all foreign substances, and prevent the retention of the buccal secretion on the surface of the exposed dentine, and attain sufficient room for a free command of the instruments to be employed in the progress of the operation; and the exposure of the diseased part of the tooth, and the surrounding surface, to plain and unmistaken vision. The decayed cavity must be enlarged, until all the disease is removed, and the surrounding walls of the cavity be supported by dentine and enamel which has not lost its vitality by the destruction of any part of its animal net-work. The cavity should be shaped so as to institute nearly an equality of dimension at the bottom and orifice of the cavity, without any acute angles to mark the form of the same, and its depth equal to its shortest breadth. The foil should then be prepared, without compromising its condition or malleability, and introduced into the cavity prepared in accordance with the above directions, with a wedge-shaped plugger, in folds, their longest axis being from the bottom to the orifice of the cavity, and so compressed, laterally and upwards, as it is introduced, as shall bring each fold in close and compact relation with the walls of the cavity or the preceding fold, not permitting a fold to change its position, either from the side or floor of the cavity, when once located; thus continuing until the cavity will no longer admit material being thus introduced, when it must be condensed with proper shaped instruments, until it is impenetrable to all substances or chemicals, excepting chlorine and electricity. When in this state, the plug should be brought to an equal, and continuous line with the surface of the tooth, and the plug, together with the surface of the dentine exposed by the file or other instrument, should be equalized, and made to receive a polished surface, by employing the usual method adopted by goldsmiths for equalizing the surface of cast gold.

Without an observance of the above general features of *care*, no operation in plugging can be relied on, with any degree of certainty in relation to its permanent beneficial influence. From from facts known, I would offer it as an opinion, that full two-thirds of the failures in the operation of plugging, is dependent on the unfaithful manner in which operators prepare the decayed cavity—leaving some part of the surrounding dental structure, that is to support the plug at its side or floor, either disorganized, or deprived of its vitality by destruction of its animal tissue, and suspension of its circulation, thus easily subject to the action of chemical agents, or the improper condensation of the material, not rendering it impermeable and compact as the ossific dental tissue.

But the report would desire not to be misunderstood as saying that operations, when completed agreeably to the above urged cautions, will always be rendered permanent and useful, during the future life of the patient. The operations must necessarily be subject to many uncertainties, as all operations connected with living tissues—the operations of which must be subject and dependent on the changes of the organized whole. Many members of the profession, forgetting this, and relying on the perfection and completeness of the manual execution of the operation, or a want of scientific observation, assure their patients, in the strongest language, of the permanency of a plug in a decayed tooth, which, from the very nature of the case, could not be productive of any thing more than temporary benefit, not from a want of completeness in the performance of the operation, but the state of the organ, from disease or formative organization, and unhealthy dependence on an enervated system.

Such a course of practice by members of the profession, who practice the duties of this branch of the healing art only mechanically—not resting its demands, and its permanent success on deductions drawn from physiological and pathological developments and indications—can occupy a position in relation to his patients, is only equalled by the physician, (but more ruinous to the profession,) who renders his professional practice subservient to *nosology* without *diagnosis*, and prescription without indication.

A dental operator who introduces plugs into the teeth of a young and enemic patient, possessing a mucous temperament, and a predisposition to inflammatory action; the mineral ingredients of the dental tissues small in quantity, and poor in quality, as shown by the complexion of the organs, character of their decay, and the firmness and density of their tissue, and assures his patients of the permanency of such operations, from a consciousness of the mechanical perfection of such operations, and a want of physiological and pathological comparison of the parts, on which all his operations rest, does as much to destroy the effort to elevate the profession, as any class of men. And this almost total neglect of logical analysis, and a tendency, when generalization is at all consulted, to speculate in fanciful analogies, and purely mechanical solutions, rather than to extract truth from facts, may be enumerated as one of the most serious defects of dental practice, and the present system of dental education, as generally patronized.

This feature of the profession carries with it a fatal influence to a successful study of disease and dental practice, by rendering it, in the estimation of the student and such a practitioner, the success of dental practice the subject of accident; and leads to disgust, and disregard of the honor and honesty of the calling; and if not an abandonment of the profession, its disgrace, and an entire absence of moral confidence in the profession, and its honorable and successful members, which experience imparts to the philosophical dentist. Again, it injures the profession, by establishing expectations, which are to be disappointed by the experience of the patient, who finds his only relief, in the recital of his misfortunes, resulting from what *he feels* to be *malpractice*, and the establishment of a want of confidence, and an absolute distrust in the community, in relation to the protective and remedial influence of dental practice over the diseases of the teeth. It also exerts a most powerful influence, in destroying harmony in the profession, and a disregard of professional ethics—a fact which is too apparent to demand illustration.

It is hoped by your report, that an attempt may be made to supply the deficiency thus briefly disclosed, in the education and practice of the profession; and trust that it will not be thought untimely for the report to urge the association, to adopt some measure that shall have in design the cultivation of proper and correct information and reasoning, upon this particular department of the profession, not only with the fellows of this Society, but the profession generally.

Dental surgery can only be faithfully, and even honestly dispensed, with a high practical excellence in its mechanical manipulations, guided and resting upon a knowledge of the development and functions of the human economy, during both health and disease. If unacquainted with the tissues, compopositions and relations of the organs of the body, how is a practitioner to understand their function? how compare the healthy with the morbid structure? how explain the physiological workings of the system? how study correctly the pathology of disease, without a previous knowledge of physiology? Without a knowledge of pathology, how determine the diagnostic symptoms and prognosis of disease? Deficiency in any of these branches, renders the practice of dental surgery downright empiricism; but with such knowledge, we may pass from the known to the unknown, and by the education of observation, are enabled to determine not only the remedial indications of our art, but determine the progress of the disease, and the tendency of its end, in spite of remedial efforts to stay its progress, and enable us to anticipate results, and by our advice be the agent in causing the patient to institute *hygienic* measures, which shall defer the day of evil, and contribute to the comfort of the sufferer, and tend to the success of the efforts of the operator.

The report would retain the attention of the Society for a moment, with the question of how great a number of plugs may be introduced in the crown of a tooth, without contributing to the susceptibility of the organ to the attacks of dental caries? In answer to this question, Koecker, in his Dental Surgery, observes: "Not unfrequently, from ten to twenty teeth may be preserved by this operation in the same individual;" but that a much greater number of teeth are often plugged, and preserved through life, than that named in the quotation, is familiar to every observing dentist; but then to what extent it renders these organs susceptible to decay, is not so satisfactorily ascertained; but that the dental organs are so influenced, cannot be doubted. The whole process which constitutes the operation of plugging a tooth, is such as tends to institute inflammatory action. If the inflammation is only slight, and resolution is followed, by the original structure of the parts having undergone no change, then the operation, (if its mechanical execution has been complete,) does not increase the susceptibility of the teeth to the attacks of dental caries; but if the inflammation assumes an ossific character, as is more frequently the result of an operation for the removal of dental caries embracing a large portion of the tooth, with patients in whose constitution the sanguineous predominates, and whose age places them near or past the meridian of life; the dental tissue loses its usual whiteness, and assumes a peculiar yellowish tint, which is often very perceptible in a tooth that has been the subject of the operation of plugging at a number of points of location on its crown, and in proximity with organs that have not been the subject of dental disease and operations, and in the mouth of the same patient, which invites comparison. Teeth subjected to such a condition, from the influences of the causes named, become also more brittle and friable. These changes are produced by certain alterations which take place in the anatomical constituents of the teeth, by the increased density of the organs, and the obliteration of many of their vessels, followed by a diminished circulation in the same.* At this point of the argument, the query arises, if the increased density of the organ would not also increase its power in resisting the attacks of dental caries? To this your report would answer, that if the increase of density of the tooth resulted from a deposit of the phosphate of lime, the query would assume a more forcible position; but arguing from analogy and experiment, the new deposit consists merely of the carbonate of lime. But even admitting the increased ability of the more compact dental tissue to withstand the attacks of corrosive agents, when considered independently, is more than counterbalanced, when considered in connection with the loss of vitality and vessels of nutrition in the dental tissue, as its density is increased. Life or vitality is in opposition to decomposition, and there is a continual struggle between the two for the dominion over matter, and just so far as an organ is deprived of its vitality and nutritive function, is it rendered an easy prey to the decomposing action of chemical agents.† An illustration of this truth is seen in the resistance which the fangs of the teeth offer to dental caries, over that of the crowns of the teeth, from their superior endowment of vitality or organization.‡

If the patient be young, and the relative proportion of earthy matter of the dental tissue be small, the inflammation is more liable to result in the destruction of the vessels of the dentine, immediately in the vicinity of the source of inflammation, and the consequent loss of its circulation, without a deposition of calcareous ingredients, which adds to the density of the organ; hence, the effect is such as to render the parts much more easily acted on by corrosive agents, than when the ossific inflammation is the result of plugging. In patients where the effect of the operation of plugging, is attended with results like those just named, and the operation be multiplied on the crown of any

* Gross' Pathological Anatomy, Boston ed. of 1839, vol. ii, p. 178.
† Müller's Physiology, vol. i, p. 86.
‡ Tome's Lectures, American Journal of Dental Science, vol. viii, p. 210.

one organ, the tooth will not only be rendered friable, and the perpendicular edges of the cavity will easily crumble and detach itself by slight violence from the surrounding structure; but the complexion of the organ will change from a polished pearly appearance, or translucent opacity, to a dull azure or a muddy opaque appearance, and frequently variegated, with light brown spots; and the enamel exhibits an irregular abraded appearance, more or less, according to the extent of the destruction of its vitality, and the action of corrosive agents on the decomposition of its surface.

With these facts, it is rendered certain, that a tooth having the operation of plugging performed for the arrest of dental caries in its structure, is rendered more vulnerable to the attacks of decomposing agents, than an organ uninfluenced by caries; although the original disease may have been completely removed by the operation which instituted a structural change in the organ, the extent of which is governed by the multiplied frequency of the operation on the same organ, and the age and health of the patient, together with the organization of the tooth operated on. To the observing and reflecting dentist, such facts are made valuable, not only in themselves, but in determining the progress of dental disease, and in deciding the character and end of operations, as well as affording valuable hints to the operator in deciding and insisting on the use of proper hygienic measures by the patient, in rendering the dentist's services valuable to the fullest extent.

It has been a question often proposed by the fellows of this association, and the profession generally, what course should be adopted, when it becomes necessary to expose the nerve or pulp cavity, in preparing the cavity of a tooth formed by caries? In answer to this question, your report will consider the various means proposed.

### *Plugging over an Exposed Nerve.*

Koecker in his Dental Surgery, describes his method of treating cases, where the decay compels the operator to expose, in the removal of the decomposed bone of the cavity, the nervous pulp; to consist after the parts have been excavated of disease, by cauterizing the exposed portion of the nervous pulp, with a heated iron wire, brought to the shape and size which will permit the operation to be performed with the greatest facility. He then completes the operation by laying a plate of lead-foil over the exposed cicatrization, believing that this metal is less liable to give rise to inflammation than any other, when wounded parts are exposed to its irritation; and then proceeds to plug the cavity by packing the gold on the cap of lead-foil in the usual way.*

The evident design of the early steps of this operation is, to lessen the volume of the nervous pulp at the point of exposure, by the peculiar contraction which attends the application of the actual cautery to the soft tissues; and also produce artificial cicatrization. The result of this method of treating the dental nervous pulp, under the circumstances now bearing discussion, we are left to suppose has generally been attended with success, while in the hands of its author, Dr. Koecker; but similar results would not appear to have been the result and experience of other members of the profession, who have attempted its practice. Inflammation seems to have followed, together with a destruction of the nervous pulp, in a much greater number of attempted cases, than success. Whether this was from a fault in making the attempt upon organs which had already past that state of health which would invite an operation, and the nervous tissue had already undergone a pathological change, which was only hastened by the operation; or a want of dexterity in the nice manipulations which mark the various stages of the operation. It is almost impossible for the operator to sustain the head of the patient with firmness sufficient, even when receiving the co-operation of a confiding patient, to cauterize only the part described, and just to the extent demanded, without implicating the surrounding parts, which would institute destructive inflammation. This fact Dr. Koecker seems to feel a conscious-

* Koecker's Dental Surgery; American Society's Edition.

ness of, for he observes that the cautery should not "penetrate deeply into the nerve," as it "would inevitably bring on suppuration and destruction of the whole membrane."

This has been so frequently, and I may say universally the result of the operation in this country, that the profession have almost wholly abandoned this method of practice, endeavoring to attain like desired results by other means, which the report will now proceed to draw the attention of the Society to the consideration of their merits.

### *Capping the Exposed Nerve with a Gold Plate.*

After the cavity has been properly prepared, by the surrounding parts exhibiting a healthy structure, if the nervous pulp of the tooth has been wounded in the operation, sufficient time is allowed, and protection offered for nature to complete cicatrization, and then a small cup-shaped plate of lamellated cast gold is fitted to the floor of the cavity—the convexity of the golden cap looking outward towards the orifice of the cavity, thus leaving a chamber between the gold cap and the nervous pulp of the tooth, not subjecting the latter to the irritation of the former; and then filling in the gold plug in the usual way. This method of operating in cases like the one under consideration, has been adopted with almost as great a variety of results, as the number of practitioners in whose hands it has been attempted. The liability of the gold capping to change its position during the operation of introducing and condensing the gold, thus giving rise to irritation and inflammation of the nervous pulp, has subjected the operation to great distrust, and by some most excellent operators to its total abandonment.

The substitution of a few coils of gold wire, from the end of a spiral spring, as used by Dr. S. P. Hullihen of Wheeling, Va., possesses much superiority over the gold plate formed into a cap for the protection of the exposed surface of the nervous pulp, from the contact of the plug with the same. The protection offered by this method, if the operation is conducted with care, is complete, and the operation rendered more successful from the exposure of its various stages to vision; and the unchanging position in which the coil of gold wire may be secured, from its pliability and easy reduction of form; and even with the cautious, little could be hazarded in pronouncing this method as contributing no little to the success of a well chosen operation.

### *Arching a Plug over an Exposed Nerve.*

Another method has been proposed for securing an exposed nerve from the irritation of a plug for the preservation of the crown of a tooth, by Prof. C. A. Harris, and practiced by him and some others of the profession, with a great deal of success, in the cases chosen for the operation. The operation differs from those named above, by so introducing the gold for forming the plug, in such a manner as shall permit its inferior surface, or that looking towards the exposed surface of the nervous pulp, to form an arch, permitting a cavity to intervene between the metal and the delicate tissue, sufficient to secure the nervous pulp from the irritation of the metallic plug.

The process by which the operation is brought to completion, may be described in a few words. The cavity in the crown of a tooth, which, from the depth of its decay, makes it necessary to expose the nervous pulp, is to be prepared in the usual way, by the complete removal of its decayed and decomposed tissue. The cavity should then be enlarged, so that a groove or excavation shall form a "shoulder" of dentine, projecting, if practicable, on either side of the exposed nerve, at the floor of the cavity; for sustaining the plug at the bottom of the same. On the approximal surface of the incisores, this injunction is often compelled to be abridged, in consequence of the frail edges of the cavity; and the loss of the surface in this direction should be gained by the additional increase of the cavity at its floor, in the direction of the long axis of the crown of the tooth. The material forming the plug must be laid in the cavity, commencing at its superior extremity, in folds, resting their interior end on the floor of the firm dentine formed by the groove, thus continuing to lay in fold after fold, using great caution that each fold be brought in absolute contact with the preceding fold, and condensing it at the same time by cautious but firm lateral pressure, that its condension may not demand as much force to be applied from without, after the cavity is filled, as well as a preventive against a change of location of the folds, after their position had been determined on, which would endanger the safety of the irritable tissue. When the folds have filled the cavity, so far as to bring the next fold of foil, if carried to the bottom of the cavity, upon the edge of the depression that borders on that part of the dentine which surrounds the exposed nervous pulp; the fold is shortened and not carried to the bottom of the cavity, and the next fold is made still a little shorter, thus continuing to shorten the folds a little, until the centre of the diameter of the exposed nerve is attained, when the folds should be gradually increased in length, to correspond with their previous shortening, until they pass the entire surface of the exposed nerve, and then brought in contact with the floor of the cavity, as in the first instance, forming a perfect arch over the nervous pulp exposed, without subjecting it to the irritation and pressure of contact. The folds of the metal should be continued after this, and ended in the usual manner, with the exception of exerting more caution than is demanded in an ordinary plug, for the condension of the same, as introduced.

The condension of the plug from without, should be commenced by gradual pressure being applied on the metal at such points, near the edge of the cavity, where the folds extend to the floor of the cavity, continuing the pressure until the consolidation of this portion is effected, and then proceed to condense, by slightly oblique pressure, applied over that part which corresponds with the arch covering the exposed nervous pulp, until the plug is ready for completion in the usual way. If, on the completion of the operation, pain does not soon succeed, or a numbness of the organ follow, showing contact or pressure of the plug on the nervous pulp, and the indications were favorable for its performance, and the mechanical execution complete, a successful issue may safely be prognosticated.

The permanent success of the operation, when either of the methods of capping an exposed nerve is adopted, depends upon the extent and character of the inflammation in the nervous pulp, which follows the proposed remedy. If it be not of an ossific character, the report hesitates not in asserting that the operation will fail of its original intent, and that, sooner or later, the destruction of the nervous pulp of the tooth will take place.

It may be laid down as an established rule, that before attempting to proceed to a successful attempt of this operation, to ascertain that the organ on which it is proposed, should not have been the subject of pain, or the nervous pulp of inflammation, or in any way changed its character or function, without a deposition of ossific ingredients.

In the young subject, both from the imperfect formation of the cavity for the reception of the dental nervous pulp, and the superior vascularity of the dental pulp, before the whole process of the formation of dentine is complete;* together with the high degree of vascularity which attend the soft tissues of the young, even if the mechanical execution be completed agreeably with the prescribed directions; inflammation very frequently succeeds, particularly if there be a predisposition to inflammation, as indicated by physical developments. If a tooth be plugged over an exposed nerve, under circumstances similar to those just described, and the nervous pulp of the tooth has perhaps been rendered irritable by the progress of the dental caries, or may not have been thus effected; the tooth, after the operation, may not exhibit any symptoms that may indicate an unfortunate termination; but if ossific inflammation does not supervene, a partial, and then a general inflammation of the nervous pulp will ensue, from the stimulus, perhaps, of some organic derangement; and an increase in size of the capillary vessels will be developed at the exposed surface of the nerve—the first point of inflammation—which will expand the tissue, until a portion occupies the chamber between the plate or cap and the nerve.

*Nasmyth's Memoirs, p. 27.

In this state, the inflammation is rarely ever visited with resolution, but the irritation is increased by the sharp edges of the bony foramina through which it protrudes into the chamber; and the returning vessels becoming strangulated by the increased volume of this part of the tissue, suppuration follows, and destruction of the vitality of the organ, so far as nutrition is effected through this medium, complete.

This being the history of most of the cases, where an unfortunate result attends the operation ; except in such cases, where the operation is attempted upon an organ, the nervous pulp of which had suffered a partial or total change in a part of its structure, it becomes an important inquiry, in a practical point, under what circumstances is the operation most likely to be attended with success, by the avoidance of the above described history of a failure.

When the decay has progressed slowly, and irritation has given rise to ossific inflammation, and when the nervous pulp is exposed, it is discovered to be lodged in a narrow fissure, formed by the new ossific deposition on either side, the indications are most favorable for success. This state of the parts is more frequently found after, or near the meridian of life, when a tendency to ossific inflammation, or earthy deposit, is instituted in the system.* The state of the health should also be consulted. Although all else indicated a successful issue, if the patient is *anemic*, or of an inflammatory diathesis, the chance of success would be greatly lessened. In like manner, the indications of success would be much increased, if the constitutional health of the patient be good, and the sanguineous predominating in his constitution, and the dental organs dense in structure.

It often happens that the dental practitioner finds the dental nervous pulp exposed, under circumstances, from its pathological condition, or from its previous destruction. When from urgent demands of the case, he is called upon to institute an effort for the preservation of the crown of the tooth, by

* Gross' Pathological Anatomy, Boston ed. 1839, vol. i, p. 341.

